# The Emerging Role of Rho Guanine Nucleotide Exchange Factors in Cardiovascular Disorders: Insights Into Atherosclerosis: A Mini Review

**DOI:** 10.3389/fcvm.2021.782098

**Published:** 2022-01-03

**Authors:** Mengqi Li, Qingzheng Jiao, Wenqiang Xin, Shulin Niu, Mingming Liu, Yanxin Song, Zengguang Wang, Xinyu Yang, Degang Liang

**Affiliations:** ^1^Department of Cardiovascular Surgery, Tianjin Medical University General Hospital, Tianjin, China; ^2^Second Department of Internal Medicine, Gucheng County Hospital, Hengshui Gucheng, Hebei, China; ^3^Department of Neurology, University of Göttingen Medical School, Göttingen, Germany; ^4^Department of Neurosurgery, Tianjin Medical University General Hospital, Tianjin, China; ^5^Department of Cardiology, Tianjin Medical University General Hospital, Tianjin, China; ^6^Department of Neurology and Immunology, Institute of Neurology, Tianjin Medical University General Hospital, Tianjin, China; ^7^Department of Nursing, Tianjin Medical University, Tianjin, China

**Keywords:** Rho GTPase, Rho GEF, atherosclerosis, mini-review, cardiovascular

## Abstract

Atherosclerosis is a leading cause of cardiovascular disease, and atherosclerotic cardiovascular disease accounts for one-third of global deaths. However, the mechanism of atherosclerosis is not fully understood. It is well-known that the Rho GTPase family, especially Rho A, plays a vital role in the development and progression of arteriosclerosis. Rho guanine nucleotide exchange factors (Rho GEFs), which act upstream of Rho GTPases, are also involved in the atheromatous pathological process. Despite some research on the role of Rho GEFS in the regulation of atherosclerosis, the number of studies is small relative to studies on the essential function of Rho GEFs. Some studies have preliminarily revealed Rho GEF regulation of atherosclerosis by experiments *in vivo* and *in vitro*. Herein, we review the advances in research on the relationship and interaction between Rho GEFs and atheroma to provide a potential reference for further study of atherosclerosis.

## Introduction

Atherosclerosis refers to the accumulation of cholesterol, and fatty, fibrous, and inflammatory substances in the arteries intima. The term atherosclerosis comes from the Greek word for “gruel” or “porridge,” reflecting the appearance and shape of the lipid material core of the typical atherosclerotic lesion (1). Approximately one-third of global deaths are attributed to atherosclerotic cardiovascular diseases (CVDs) (2). Atherosclerotic arteries lead to acute coronary syndromes, ischemic strokes, aneurysms, intermittent claudication, ulceration, and gangrene (1). Atherosclerosis is characterized by the development of lesions in the wall of the artery, and a disorder in any of the arteries is likely to be a starting factor of atherogenesis. As a main barrier of the artery, the endothelium plays a vital role in the development of atherosclerosis (3, 4). Small GTPases activated by Rho guanine nucleotide exchange factors (GEFs) partly or completely regulate endothelial cell migration and proliferation and adherens junctions of impermeable surfaces. In addition to the endothelium, inflammatory, or anti-inflammatory macrophages controlled by Rho GEFs, the small GTPase RhoA and its downstream effector, and Rho-associated coiled-coil containing kinases (ROCK) are also critical components for the development, regression, or stabilization of the atherosclerotic plaque (5). The mechanism of atherosclerosis is not fully elucidated. It is well-known that the Rho GTPase family, especially Rho A, has a vital function in the development and progression of arteriosclerosis. Rho GEFs, the upstream activators of Rho GTPase, are also involved in the atheromatous pathological process. In this mini review, we will briefly discuss the role of Rho GEFs and their potential target in atherosclerosis.

## The Essential Characteristics and Etiology of Atherosclerosis

During 2009 to 2019, CVD was the number one cause of death (222.58 deaths per 1,00,000 in 2009; 239.9 death per 1,00,000 in 2019) (6). Atherosclerosis is the leading cause of CVD worldwide (1). Globally, more than 75% of CVD deaths occur in low- and middle-income countries (7). CVDs cause 10% of disability-adjusted life years lost in low- and middle-income countries and 18% of disability-adjusted life years lost in high-income countries (8). These statistics indicate that atherosclerosis places a heavy economic burden on developing countries. Although the specific mechanism of atherosclerosis remains unknown, a consensus is that multiple factors bring about atherosclerosis (9). These factors can be divided into genetic and acquired, and they work together to drive the development and progression of atherosclerosis. From another perspective and according to the progress in research, atherosclerosis is also considered a chronic disease (10). Inflammation and lipoprotein metabolism remain the focus of atherosclerosis research (11).

**Figure 1:**
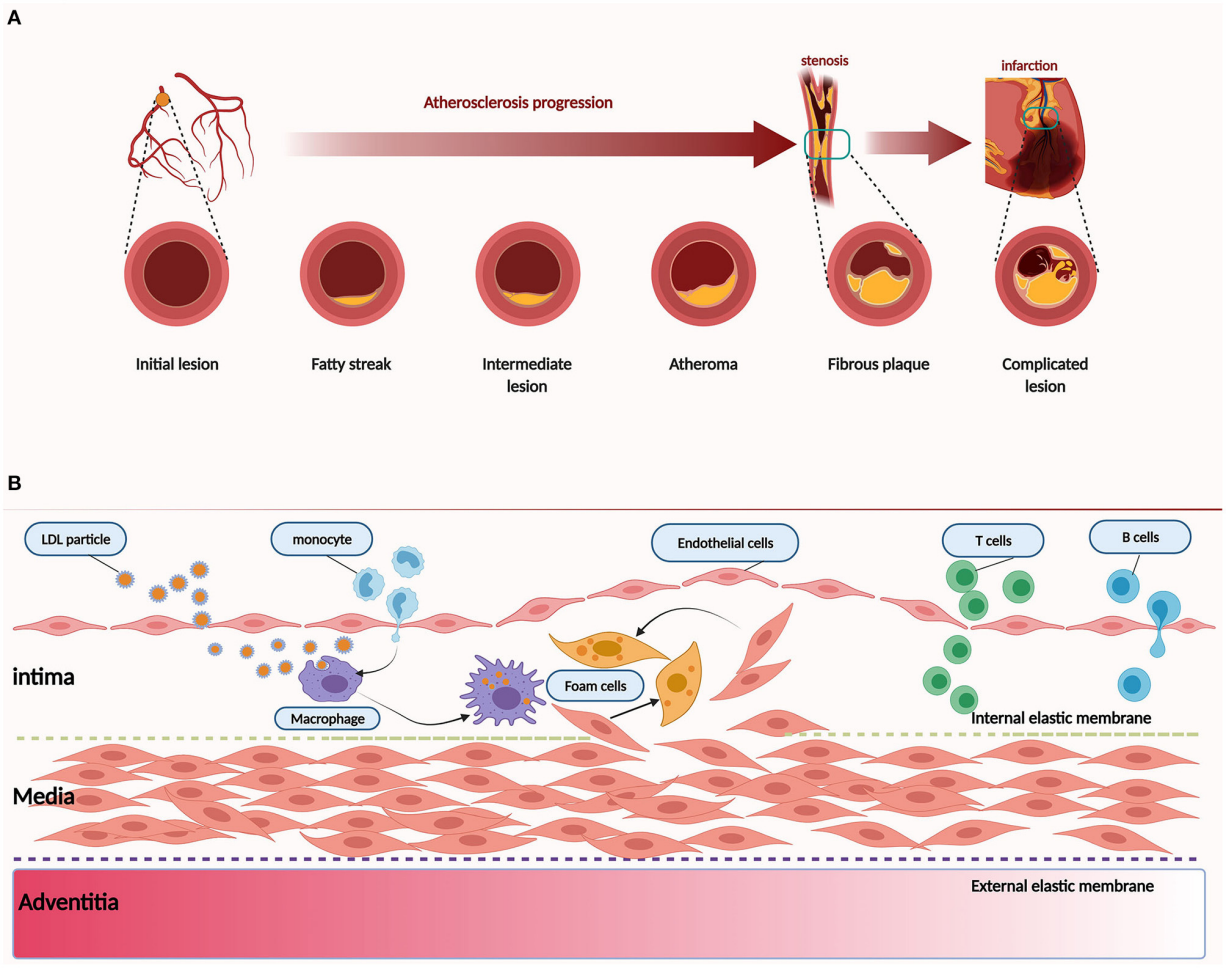
Pathology of atherosclerosis. **(A)** Atherogenesis stages. **(B)** Atherosclerosis is a dynamic process. Inflammation is associated with atherosclerosis in different stages. Dysfunction of the endothelium is a primary event in atherogenesis, which can be caused by various risk factors, such as physical stress and chemical stimulants. LDL particles accumulate and are absorbed by macrophages derived from monocytes of blood and smooth muscle cells that migrate from the media to the intima. As another critical step in atherogenesis, leukocytes, such as T and B cells, are recruited to the arterial wall to produce cytokines and direct monocytes to the atherosclerotic lesion.

Here, we provide a brief introduction to the etiology of atherosclerosis. The artery wall provides the lining and multiple factors that conspire to produce atherosclerosis (Figure 1). In addition to traditional risk factors, novel risk factors (e.g., exposure to air pollution and noise, sleep deprivation, psychosocial stress, and intestinal microbiome) could be acquired to promote and affect atherosclerosis at different stages (12). Convincing evidence from genetic, epidemiologic, and clinical studies suggests that low-density lipoprotein cholesterol (LDL-C) causes atherosclerosis (13). LDL-C is a crucial factor in the initial stage of atherosclerosis. In this stage, LDL-C fluxes into the arterial wall and is kept within the intimal layer to provoke atherosclerosis. The transendothelial movement of LDL impairs the arterial wall and barrier function of the endothelium (14). LDL particles gradually gather in the subendothelial space and attach to intimal proteoglycans. The smooth muscle cells and macrophages become engulfed with lipid and contribute to foam cell formation and lesion development (15). In the progression stage, smooth muscle cells that migrate from the media into the intima as well as monocyte-derived macrophages accumulate under the artery lining, and foam cell numbers increase as the atherosclerotic plaque grows (16, 17). Macrophages and smooth muscle cells activate programmed cell death, which accumulates in the lipid-enriched nidus or necrotic core (18). During the development of atherosclerosis, calcification lesions form and accumulate in many arterial regions (19). The mechanism of Rho GEF involvement in atherosclerosis will be described in section The Emerging Role and Interaction Mechanisms of Rho GEF in Atherosclerosis.

## Molecular Character of Rho GEFs

The current research shows that the human genome of the Rho GEF family encodes 20 GTPase and 82 RhoGEFs (20). There are two groups of Rho GEFs, dedicator of cytokinesis (DOCK)-related proteins and diffuse B-cell lymphoma (Dbl)-like families (21). In humans, Dbl-like families are the most prominent Rho family GEFs, which comprise 71 members and can be structured in 20 subfamilies (22). Sharing a 170–190 amino acid Dbl homology (DH) domain is the common characteristic of Dbl-like Rho GEFs. Most Dbl-like Rho GEFs have a conserved core that is composed of DOCK-homology region (DHR)-1/C2 and DOCK homology region 2 (DHR2) domains, either single or connected with single SH3 or pleckstrin homology (PH) domains (21). Other than the DH domain, Dbl-like Rho GEFs contain domains that mediate interaction with membranes, proteins or phosphorylated amino acids, PH domains, or diverse enzymatic activities such as kinases, phosphatases, GEFs, or GAP. All known DH domains of Rho GEFs have related C-terminal PH domains involved in targeting and regulatory functions. The active site of DH domains is located near the junction between the DH domain and the PH domain (23). The DH and PH domains work in tandem, which allows the catalysis of Rho proteins (24). DOCK proteins are unrelated to the Dbl family in structure and mechanical properties, affecting Cdc42 and Rac, but not Rho A. The characteristics of DOCK proteins are a conserved catalytic domain (DHR2) and a phospholipid-binding domain (DHR1), which allow the GEFs to target the membrane (25).

## Rho GTPases: The Molecular Functional Performers of Rho GEFs

**Figure 2:**
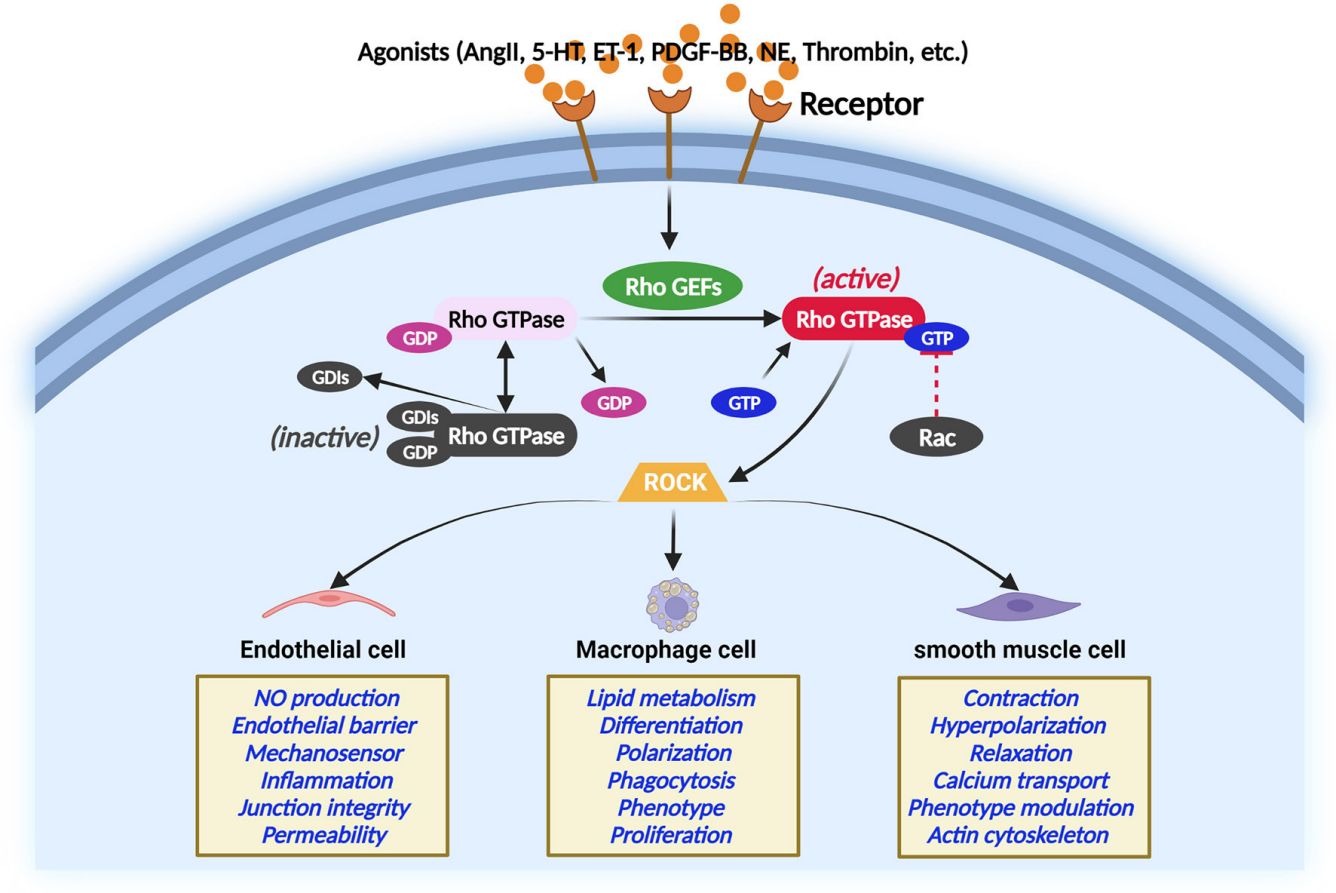
Regulation of Rho GTPase in different main atherosclerosis-related cells. Rho GTPase acts as a molecular switch that cycles between an inactive and active GTP-bound conformation interacting with ROCK. The activity of Rho GTPases is controlled by Rho GEFs that catalyze the exchange of GDP for GTP. In contrast, GTPase-activating proteins stimulate the intrinsic GTPase activity and inactivate Rho GTPase. Guanine nucleotide dissociation inhibitors block spontaneous Rho GTPase activation. The Rho GTPase/ROCK pathway plays important role in the main atherosclerosis-related cellular functions.

Rho GEFs activate Rho GTPases by catalyzing the exchange of GDP for GTP (26). RhoGEFs, RhoGAPs, and RhoGDIs work together to regulate Rho GTPases. During this course, GTP-bound active conformation and GDP-bound inactive conformation of Rho GTP form a cycle that coordinates the activity of Rho GTP to activate effector proteins and elicit a cellular response (27). In response to extracellular stimuli, many Rho GEFs interact with specific proteins that Rho GTPases target and coordinate the Rho GTPase signaling network at specific sites in cells (28). The Rho GTPase regulation and Rho GTPase function in different vascular cells are illustrated in Figure 2 (29–33). A noted example is a complex comprising the Rho GEF β-PIX (RAC/CDC42-specific) with the RAC/CDC42 acting on PAK, which conducts turnover of integrin-containing focal adhesions (28).

Another example is the P-REX1, which brings RAC1 together with its effector FLI1 to promote cell migration (34). Information regarding this aspect, however, is scarce. Rho GEF proteins make up a complex interactive network that accurately activates Rho GTPases in translational modifications (35). In the status of the active conformation of Rho GTPases, the Rho GTPases interact with a variety of effector proteins, including kinases, adaptor proteins, and actin regulators, thereby activating cellular responses and leading to changes at the cellular level, which depend on the stimulus effectiveness and cell type (35).

## Effects of Rho GEFs on the Cardiovascular System

A growing body of evidence suggests that the over-activation of Rho proteins is the shared pathogenesis of several cardiovascular disorders such as atherosclerosis, hypertension, and diabetes (36). Rho GEFs can activate Rho proteins by catalyzing the exchange of GDP for GTP and regulating Rho protein activity (36). In addition, some Rho GEFs have been identified as susceptibility genes for CVDs. Using next generation sequencing (NGS), bioinformatics, and ARHGEF17-deficient zebrafish, our research group found that ARHGEF17 is a candidate gene for intracranial aneurysm (IA) (37). In the aforementioned study, rs2298808 of ARHGEF17 was shown to be related to IA in the Chinese cohort, but arhgef17 knockdown of zebrafish caused bleeding and endothelial lesion in the brain region. Some Rho GEFs are more expressed in rat aorta and mesenteric arteries than in other arteries, such as PDZ-Rho GEF at mRNA and protein levels (38, 39). Vascular smooth muscle cells (VSMCs) and vascular endothelial cells are vital components of the artery, and regulation of the functions of these two types of cells by GEFs may be a participant in artery function. Angiotensin II (Ang II) regulates VSMC function by inducing tyrosine phosphorylation of Vav. PSD95-discs large-ZO1 (PDZ)-Rho GEF has been shown to be tyrosine-phosphorylated by temporary stimulation with Ang II (40). However, whether Rho GEFs are involved in the Ang II-regulated regulation of VSMCs remains unknown. Tumor endothelial marker-4 (TEM4) can also be called ARHGEF17 or p164-RhoGEF, and it regulates the integrity of the intercellular junctions and endothelial cell function (41). Rho GEF TEM4, which supports the persistence of cell migration by adjusting actin fibers and cell adhesions in protruding membranes, regulates the migration of endothelial cells.

## The Emerging Role and Interaction Mechanisms of Rho GEF in Atherosclerosis

### Current Status of the Relationship Between Rho GEFs and Atherosclerosis

The authors retrieved data from PubMed and Google Scholar with “Rho GEFs,” “guanine nucleotide exchange factor,” and/or “atherosclerosis” as the search terms to look for direct evidence of Rho GEF regulation of atherosclerosis after screening and refining the data. The search revealed some research on the role of Rho GEFs in atherosclerosis regulation; however, the number of studies is small compared with the research on the essential function of Rho GEF (Table 1). Based on experiments *in vivo/vitro*, several studies indicated that Rho GEFs regulate atherosclerosis. Samson et al. found that RhoG guanine nucleotide exchange factor SGEF (Arhgef26) gave rise to the formation of ICAM-1-induced endothelial docking structures, which promote white blood cell transendothelial movement, enter arterial walls, and advance atherosclerosis (42). In SGEF-deficient mice crossed with ApoE null mice, which were fed a Western diet, the level of aortic atherosclerosis of SGEF-deficient mice was reduced. In this work, the authors demonstrated that SGEF facilitates endothelial docking structures, and thus leukocytes increase at athero-prone sites of inflammation-associated high shear flow (42). In addition to SGEF, the other Rho GEF protein, RhoA GEF Arhgef1 that is essential for Ang II-induced inflammation, is also a key molecule for atherosclerosis. Rebuilding Ldlr^−/−^ mice with Arhgef1-deficient bone marrow restrained high-fat diet-induced atherosclerosis, whereas restriction of Ldlr^−/−^ Arhgef1^−/−^ with wild-type (WT) bone marrow (BW) exacerbated atherosclerotic lesion formation. Among the reasons for this finding, Arhgef1 was activated, and more leukocytes were recruited to the endothelium to accelerate atherosclerosis (43). In another study, experiments *in vivo* and *in vitro* found that the Arhgef7 (Rho guanine nucleotide exchange factor 7, beta Pix) interacted with Scribble (Scrib) to produce antiatherosclerotic functions by maintaining the endothelial barrier function (45). Rho GEF dedicator of cytokinesis 4 (DOCK4) facilitated internalization of SR-B1 and transport of LDL by coupling the binding of LDL to SR-B1 by activating RAC1. SR-B1 drove LDL transcytosis of the endothelium by DOCK4 to promote atherosclerosis (46). Double-null mice ApoE/vav1, fed a Western diet, had a clear reduction in overall aortic atherosclerotic lesion area and fewer macrophages and foam cells in the aortic sinus. Rho GEF Vavs functioned as key molecular links that integrated hyperlipidemia to proatherogenic monocyte/macrophage responses (44). P-Rex1 is one of the major Rho GEFs activating Rac1. Deficiency of P-Rex1 in mouse macrophages was found to significantly decrease macrophage chemotaxis, superoxide production (SOD), and Rac1 activation in response to chemo-attractants, suggesting the regulatory role of P-Rex1 in atherosclerosis (48). Recent work reported that CDGI is essential for atherosclerotic plaque development because it can lead to leukocyte recruitment to the lesion area (47).

**Table 1:** Direct evidence of Rho GEF regulation of atherosclerosis.

**Rho GEF name**	**Rho GTPase effector**	**Key features related to atherosclerosis**
**Experiments** ***in vivo*****/*****vitro***		
SGEF (42)	RhoG	SGEF promotes endothelial docking structures and thereby retention of leukocytes at atherosclerosis-prone sites of inflammation experiencing high shear flow.
Arhgef1 (43)	Rho A	Arhgef1 activation in leukocytes is causally associated with the development of atherosclerosis.
Vavs (44)	Rac1 and Rho A	Vavs act as critical molecular links coupling hyperlipidemia with proatherogenic monocyte/macrophage responses.
Arhgef7 (beta Pix) (45)	Rac1 and Cdc42	Arhgef7 interacts with Scrib to maintain endothelial barrier function and normal vascular permeability.
DOCK4 (46)	RAC1	DOCK4 promotes internalization of SR-B1 and transport of LDL by coupling the binding of LDL to SR-B1 with activation of RAC1. The expression of DOCK4 is increased in atherosclerosis-prone regions of the mouse aorta before lesion formation, and in human atherosclerotic arteries when compared with normal arteries.
CDGI (47)	Rap1	CDGI contributes to platelet-leukocyte aggregate formation and leukocyte recruitment to the atherosclerotic lesion area.
P-Rex1 (48)	Rac1	P-Rex1 regulates Rac1 activation and chemotaxis in macrophages, and may be a regulator for atherosclerosis.
**Genetic research**		
MCF2L (49)	The rare functional variant [c.2066A4G p. (Asp689Gly)] in MCF2L, leading to impaired DH function, was identified in a small pedigree with premature CVD. The presence of MCF2L in human atherosclerotic plaque specimen lends further support to its potential role in atherosclerosis.	
ARHGEF10 (50)	Rs4376531 polymorphism in the ARHGEF10 gene is a risk factor for AS in the Han Chinese population.	
KALRN (51, 52)	Peakwide mapping on chromosome 3q13 identifies the KALRN as a novel candidate gene for coronary artery disease. The GG genotype and the G allele of rs9289231 polymorphism of KALRN were found to be genetic risk factors for CAD in an Iranian population, especially in early-stage atherosclerotic vascular disease.	
DOCK7 (53)	The DOCK7-ANGPTL3 SNPs and their haplotypes were associated with the angiographic severity to coronary artery atherosclerosis and the risk of CAD and IS in the Southern Chinese Han population.	

In addition to experiments *in vivo/vitro*, several forms of genetic research have shown the relationship between Rho GEFs. A rare functional variant [c.2066A4G p. (Asp689Gly)] in *MCF2L* was detected in a small pedigree with premature CVD. The presence of the *MCF2L* protein was found in human atherosclerotic coronary arterial tissue compared with healthy tissue, and the variant led to impaired MCF2L-DH-domain-dependent actin stress fiber formation, indicating that *MCF2L* might play a role in premature atherosclerosis pathobiology (49). A genetic finding suggested that the rs4376531 of *ARHGEF10* is a risk factor for atherothrombotic stroke in the Chinese Han people (50). Associations were found in SNPs in the *KALRN* gene from the Rho GEF family and CDGAP and MYLK from the Rho GTPase-signaling pathway, suggesting the importance of Rho GEF KALRN in atherosclerotic pathogenesis (51, 52). The DOCK-ANGPTL3 SNPs (rs12563308, rs12563308, and rs1748195) and their haplotypes (rs1748195G-rs12563308T) were found to be related to the severity of coronary artery atherosclerosis in the Chinese Han population (53).

### Mechanisms of Atherosclerosis Regulation by Rho GEFs

Rho GEFs localize to the cell membrane where they cause activation of the Rho-GTP (35). Pro-inflammatory and anti-inflammatory macrophages are critical factors for the expansion, progression, or steadying of atherosclerotic plaque (5). Rho-GTP proteins, especially RhoA, control this process. Rho GEF is upstream of Rho-GTP, and the specific mechanism by which Rho GEFs regulate atherosclerosis by Rho-GTP remains unclear and needs further study. However, some Rho GEF proteins may be involved in the occurrence and progression of atherosclerosis, and in the composition of atherosclerotic arteries, including the endothelium, smooth muscle, and macrophages (36).

#### Regulation of RhoGEFs on Endothelial Cells

Endothelial cell dysfunction is essential to the pathobiology of CVD (54) and some Rho GEFs are involved in this process. Tiam1 is a specific Rho GEF for Rac1 and increased permeability by damaging intercellular junctions between the endothelial cells (55). The Rac-specific GEF P-Rex1 acted downstream of TNFα to convey endothelial barrier disruption (56). Another Rho GEF, Itsn2L, which is specific for Cdc42, displayed a specific subcellular localization, regulated caveolae endocytosis, and interacted with the actin network in endotheliocytes (57). Knockdown of RhoA GEF-H1 *in vitro* contributed to a rise in the endothelial permeability and actin stress fiber formation, suggesting that GEF-H1 is required to maintain the balance between endothelial permeability and barrier integrity (58). However, AGE and its major constituent, S1PC, inhibited the phosphorylation and activation of GEF-H1 to protect the endothelial barrier through the protection of junctional proteins on plasma membranes (59). Klems et al. used zebrafish embryos and endothelial cell models to show that Rho GEF Trio controlled the formation of enlargement and extension of arterial endothelium. It activated Rac1 and RhoG in the cell periphery, bringing about F-actin cytoskeleton altering of myosin-based tension at regions of cell junction focal adhesions (60). Recently, it was reported that Rho GEF ITSN1 interacted with RhoJ to promote endothelial cell sprouting (61). In the aforementioned work, removing RhoGEF17 disrupted cell-cell and cell–substrate interactions, preventing cell death and inhibiting cell growth in the endothelial cell (62). All of the aforementioned works indicate that Rho GEFs may be involved in the atherosclerotic process; nevertheless, further experiments are needed to verify this assumption.

#### Regulation of RhoGEFs on VSMCs

The proliferation and migration of VSMCs to the subendothelial layer are some of the features of atherosclerosis (16). Several Rho GEFs participate in arterial smooth muscle cell proliferation. For instance, one work showed that the Rho GEF Kalirin, which activates Rac-1 and RhoA, is raised in early atherogenesis and promotes arterial SMC migration and proliferation *in vitro* and *in vivo* (63). MicroRNA miR-27a-3p was found to suppress ARHGEF26 (also known as SGEF) and inhibit SMC proliferation (64). LARG, a RhoA-specific Rho GEF, regulated SMC migration and stress fiber formation (65). One study found that the downregulation of RAP1GDS1 (SmgGDS) gave rise to deceased activated RhoA levels, higher cell spreading, and reduction in the characteristic stretched morphology of VSMCs (66). SmgGDS was also shown to be a regulator of myosin arrangement and contraction for VSMCs (66). Another study found that Rho GEF Vav3 regulated VSMC proliferation and migration by motivating Rac1/PAK signaling, which appears to be a new potential therapeutic target to inhibit vascular proliferative diseases (67).

#### RhoGEF Regulation of Macrophages

Macrophages are a fundamental contributor to atherosclerosis and can be affected by Rho GEFs (68). Studies show that directly inhibiting Rho-GEFs can diversely affect M0, M1, and M2 with Y16 (Rho GEF DH–PH domain blocker) and Rho sin (Rho GEF-binding domain blocker) (69). This indicates that CD36-mediated macrophage foam cell formation and CD36-dependent uptake of oxLDL can be regulated by the Vav family Rho GEF Vav-1,−2, and−3 (70). It also suggests that the CD36/Vav signaling pathway is required for the macrophage foam cell formation (70). By methods of siRNA-mediated silencing, pharmacological inhibition, genetic knockout, and stable overexpression, one work elucidated critical roles for Cdc42 and Vav in promoting actin polymerization during the formation of the lysosomal synapse (71). The study found that in the course of lysosomal synapse formation, catabolism of aggregated LDL and foam cell formation, and active macrophage F-actin reorganization were also regulated by Vav (71). The macrophage inflammation responses could be related by Rho GEF BIG1, and the downregulation of BIG1 induced by LPS mainly is related to TLR4 signaling in THP-1-derived macrophages (72). These aforementioned studies disclose that Rho GEFs can affect macrophages during the atherosclerotic process.

### Regulated Function of Rho GEF/Rho GTPase/ROCK in Atherosclerosis

**Figure 3:**
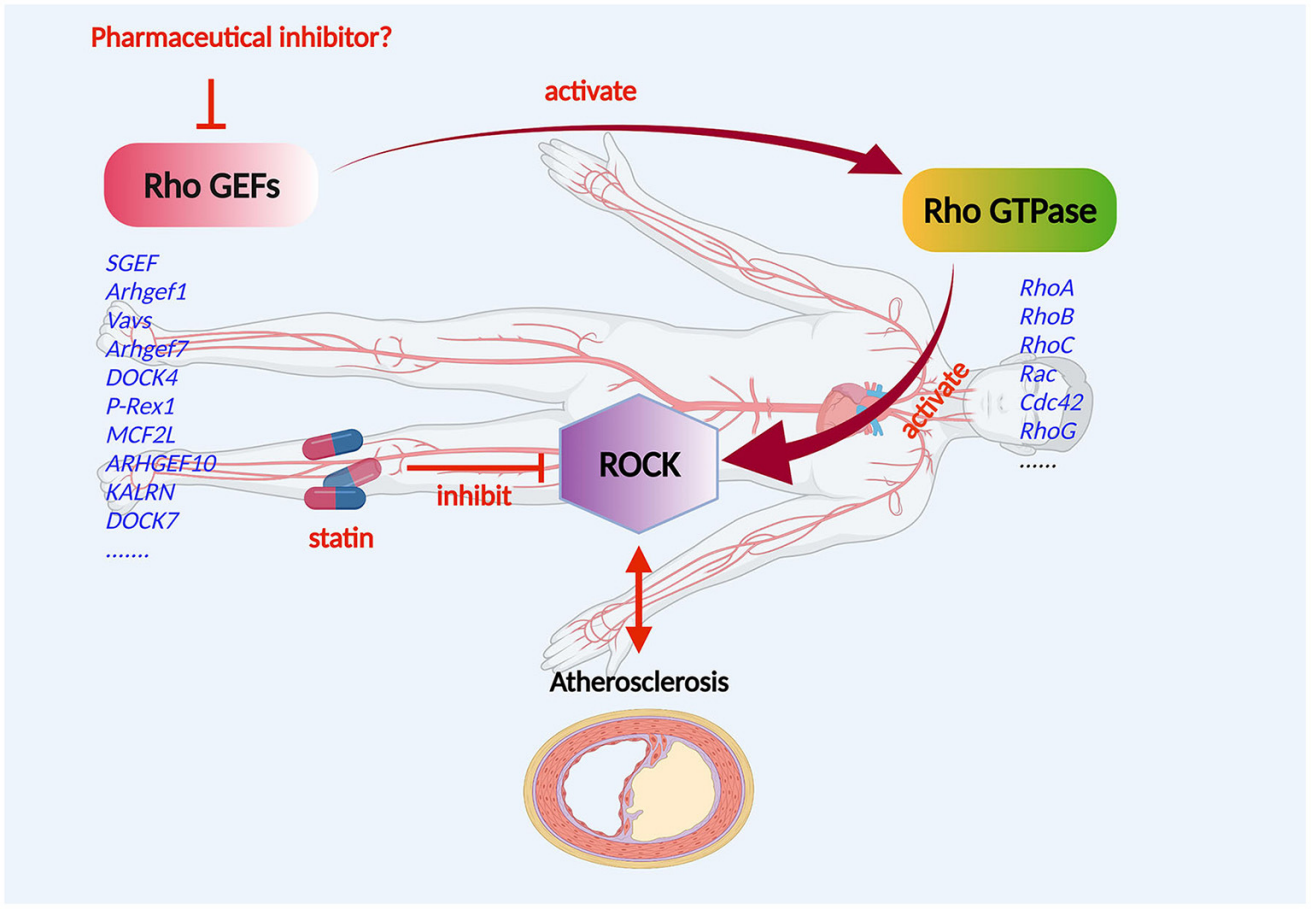
Mechanism of Rho GEF regulation of atherosclerosis. Rho GEF proteins may be involved in the occurrence and progression of atherosclerosis, and the composition of atherosclerotic arteries including the endothelium, smooth muscle, and macrophages. The Rho GEF/Rho GTPase/ROCK signaling pathway plays an important role in the development of atherosclerotic disease. Rho GEFs, as activators and the most direct upstream molecules of Rho proteins, are expressed in cardiovascular cells and are suitable candidate targets for the drug therapy of atherosclerotic disorders.

The Rho GEF/Rho GTPase/ROCK signaling pathway is vital for the development of CVD (Figure 3). In the progression of atherosclerosis, ROCK should be regarded as a pro-inflammatory and proatherogenic molecule that promotes atherosclerosis (73). In the Rho GTPases, Rho A and its primary effector, ROCK, play a central role in the cardiovascular system (74). Studies showed that disturbed blood flow, which causes endothelial dysfunction by the Rho A/ROCK signaling pathway and mechanotransduction mechanism, leads to the advancement of atherosclerosis (75, 76). The distinguishing features of the atherosclerotic artery are proliferation, phenotype modulation, and the redox state of VSCMs (77, 78). Significantly, Rho-kinase is extensively involved in this pathological process (29). ROCK activity of leukocytes in atherosclerotic patients was found to be increased (79), indicating that ROCK activity may be a surrogate marker for patients with atherosclerosis. The Rho A/ROCK signaling pathway could be inhibited by endogenic nitric oxide, which might indicate key crosstalk of ROCKs with the endothelial function (80). As essential drugs, statins target the inhibition of the Rho/ROCK pathway to reduce atherosclerosis and possibly CVD (81). However, the effects of Rho GEF/Rho GTPase/ROCK on endothelial cells, inflammatory cells, fibroblasts, and VSMCs can boost atherosclerosis, which may be responsible for the pleiotropic effects of statins (82).

## Prospects and Conclusion

Without a doubt, Rho proteins have a significant effect on the cardiovascular system (83, 84). Rho GEFs, as activators and the most direct upstream molecules of Rho proteins, are expressed in cardiovascular cells and are suitable candidate targets for the drug therapy of atherosclerotic disorders (Figure 3).

Some drugs targeting Rho proteins require complex approaches or processes to treat atherosclerosis, such as statins, which require multiple steps to lower LDL (82). In this process, the more steps and multiple effects that are involved can cause more side effects. Currently, although statins have a very positive effect, various statin-associated symptoms, including statin-associated muscle symptoms, diabetes mellitus, and central nervous system complaints, have been reported (85). Statins competitively and reversibly inhibit HMG-CoA reductase by their lactone ring and side chains that help them bind to the enzyme's active site (86), which contributes to the inhibition of cholesterol synthesis and brings about decreasing cholesterol production and upregulating LDL receptor (87). Statins can reduce the ROCK activity of white cells independent of LDL reduction (88). In human aortic endothelial cells, statins delay tissue factor induction by thrombin in a Rho/ROCK-dependent manner (89). Regulating the Rho/ROCK pathway with Rho GEFs could achieve the same therapeutic effect as statins while avoiding its many side effects. Hence, the authors suggest the regulated target of Rho proteins by Rho GEFs as a potential therapeutic target in atherosclerosis disease.

Compared with the current drugs targeting Rho proteins, the use of Rho GEFs to directly regulate the Rho proteins will be more effective and have fewer side effects. This progress in cardiovascular medicine is a sterling example of how the clinical application of scientific discoveries benefits patients. The findings of current research open a door of understanding into the development of atherosclerosis. The discovery of prosaposin as a potential therapeutic target may lead to the development of new therapeutics, which is precisely what precision medicine advocates (90). Of course, more research on Rho GEF regulation of atherosclerosis is needed.

## Author Contributions

DL, XY, and ZW designed and conceptualized the article. MLi and WX collected material and prepared the manuscript, figures, and tables. All authors significantly contributed to the writing of the manuscript and provided important intellectual content.

## Funding

This study was supported by (1) the Youth Project of National Natural Science Foundation of China (No. 82000477); (2) Tianjin Key Research and Development Plan, Key Project of Science and Technology Support (No. 20YFZCSY00010); (3) Scientific Research Program Project of Tianjin Education Commission (No. 2018ZD03); and (4) the Natural Science Foundation of Tianjin, China (Grant No. 20JCZDJC00300).

## Conflict of Interest

The authors declare that the research was conducted in the absence of any commercial or financial relationships that could be construed as a potential conflict of interest.

## Publisher's Note

All claims expressed in this article are solely those of the authors and do not necessarily represent those of their affiliated organizations, or those of the publisher, the editors and the reviewers. Any product that may be evaluated in this article, or claim that may be made by its manufacturer, is not guaranteed or endorsed by the publisher.
