# Corrigendum: The Emerging Role of Rho Guanine Nucleotide Exchange Factors in Cardiovascular Disorders: Insights Into Atherosclerosis: A Mini Review

**DOI:** 10.3389/fcvm.2022.850258

**Published:** 2022-01-31

**Authors:** Mengqi Li, Qingzheng Jiao, Wenqiang Xin, Shulin Niu, Mingming Liu, Yanxin Song, Zengguang Wang, Xinyu Yang, Degang Liang

**Affiliations:** ^1^Department of Cardiovascular Surgery, Tianjin Medical University General Hospital, Tianjin, China; ^2^Second Department of Internal Medicine, Gucheng County Hospital, Hengshui Gucheng, Hebei, China; ^3^Department of Neurology, University of Göttingen Medical School, Göttingen, Germany; ^4^Department of Neurosurgery, Tianjin Medical University General Hospital, Tianjin, China; ^5^Department of Cardiology, Tianjin Medical University General Hospital, Tianjin, China; ^6^Department of Neurology and Immunology, Institute of Neurology, Tianjin Medical University General Hospital, Tianjin, China; ^7^Department of Nursing, Tianjin Medical University, Tianjin, China

**Keywords:** Rho GTPase, Rho GEF, atherosclerosis, mini-review, cardiovascular

In the published article, there was an error in affiliation. Instead of **Zengguang Wang**^3*^ **and Xinyu Yang**^3*^, it should be **Zengguang Wang**^4*^ **and Xinyu Yang**^4*^.

The authors apologize for this error and state that this does not change the scientific conclusions of the article in any way. The original article has been updated.

## Publisher's Note

All claims expressed in this article are solely those of the authors and do not necessarily represent those of their affiliated organizations, or those of the publisher, the editors and the reviewers. Any product that may be evaluated in this article, or claim that may be made by its manufacturer, is not guaranteed or endorsed by the publisher.

